# ADAMTS Proteins and Vascular Remodeling in Aortic Aneurysms

**DOI:** 10.3390/biom12010012

**Published:** 2021-12-22

**Authors:** Zakaria Mougin, Julia Huguet Herrero, Catherine Boileau, Carine Le Goff

**Affiliations:** 1 INSERM U1148, Laboratory of Vascular Translational Science, Université de Paris, Hôpital Bichat, F-75018 Paris, France; zakaria.mougin@inserm.fr (Z.M.); juliahuguet.03@gmail.com (J.H.H.); catherine.boileau@inserm.fr (C.B.); 2 Département de Génétique, AP-HP, Hôpital Bichat, F-75018 Paris, France

**Keywords:** ADAMTS, aorta, aneurysms, human, mouse model

## Abstract

Extracellular matrix (ECM) in the vascular wall is a highly dynamic structure composed of a set of different molecules such as elastins, collagens, fibronectin (Fn), laminins, proteoglycans, and polysaccharides. ECM undergoes remodeling processes to regulate vascular smooth muscle and endothelial cells’ proliferation, differentiation, and adhesion. Abnormalities affecting the ECM can lead to alteration in cellular behavior and from this, this can conduce to the development of pathologies. Metalloproteases play a key role in maintaining the homeostasis of ECM by mediating the cleavage of different ECM components. There are different types of metalloproteases: matrix metalloproteinases (MMPs), disintegrin and metalloproteinases (ADAMs), and ADAMs with thrombospondin motifs (ADAMTSs). ADAMTSs have been found to participate in cardiovascular physiology and diseases and specifically in aortic aneurysms. This review aims to decipher the potential role of ADAMTS proteins in the physiopathologic development of Thoracic Aortic Aneurysms (TAA) and Abdominal Aortic Aneurysms (AAA). This review will focus on what is known on the ADAMTS family involved in human aneurysms from human tissues to mouse models. The recent findings on *THSD4* (encoding ADAMTSL6) mutations in TAA give a new insight on the involvement of the ADAMTS family in TAA.

## 1. Introduction

An aortic aneurysm (AA) is a dilatation that occurs in the aorta, a major blood vessel that comes out of the heart and carries blood throughout the body. Two types of aortic aneurysms can transpire: (1) abdominal aortic aneurysms (AAA), which occur in the descending aorta in the abdomen, and (2) thoracic aortic aneurysms (TAA) which occur in the aortic section of the chest cavity (ascending, cross, and early descending). Aneurysms from genetic origin usually appear in the thoracic aorta, and more specifically at the ascending aorta. With or without aneurysms, aortic dissections can also occur. A dissection is a loss of integrity between the media and adventitial layers of the aorta, which leads to the formation of a false lumen. Differing classifications of aortic dissections exist, such as DeBakey type 1, which involves the ascending and descending aorta (e.g., Stanford A), DeBakey type 2 involving the ascending aorta only (e.g., Stanford A), and DeBakey type 3 involving the descending aorta only, starting after subclavian artery (e.g., Stanford B).

Beyond cellular components such as smooth muscle cells, fibroblasts, endothelial cells, and immune cells, the aortic extracellular matrix (ECM) has a crucial role in maintaining homeostasis and the physiopathological mechanisms of thoracic aortic aneurysms and dissections (TAAD). Almost all the thickness of the aorta is made up of ECM proteins, such as fibrillar proteins (e.g., collagens, fibrillins, and elastin), proteoglycans (e.g., heparan-sulfates glycoproteins and perlecan), and metalloproteases. It must be emphasized that elastin fibers and fibrillar collagens make up to 50% of the dry weight of large caliber arteries. ECM proteins, components of vessel walls, are in permanent replacement due to a consistent turnover of proteins with the help of metalloproteases, specifically matrix metalloproteases (MMP) and a disintegrin and metalloproteases with thrombospondin motifs (ADAMTS). The hallmarks of TAA are the progressive degradation of the media due to the decreased integrity of elastic fibers, increased elastolysis activity (through metalloproteases), and TGF-β signaling pathway overactivation [1].

This study aims to decipher the potential role of ADAMTS proteins in the physiopathologic development of TAA and AAA. This review will focus on what is known from mice models, human tissues, and the first protein from ADAMTS involved in human aneurysms.

### ADAMTS Proteins

**Figure 1:**
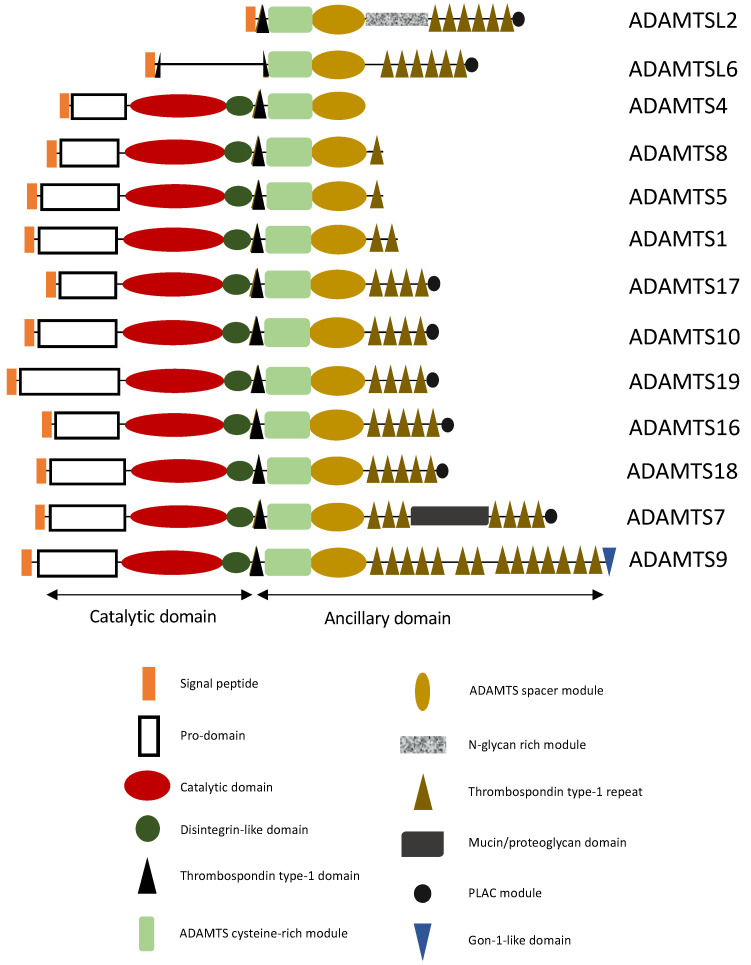
Structural representation of ADAMTS(-L) involved in aortic aneurysms. ADAMTS have a characteristic domain organization consisting of a catalytic domain at their N-terminus, and a C-terminal ancillary domain (Figure 1). The ancillary domain has a modular organization containing distinct modules, such as an ADAMTS cysteine-rich module, an ADAMTS spacer module, thrombospondin type-1 domains and the presence or absence of PLAC module and/or mucin/proteoglycan domain (see Figure).

The ADAMTS family constitutes a group of proteins composed of 19 enzymes and 7 ADAMTS-like proteins. The ADAMTS share a specific domain organization with a signal peptide, a prodomain, a catalytic domain, and an ancillary domain. Their catalytic activity involves zinc and three conserved histidine residues [2]. The ancillary domain may consist of a disintegrin-like domain, a first thrombospondin-type I motif, a cysteine-rich domain, a spacer domain, and other thrombospondin motifs. Certain ADAMTS have additional domains, such as the PLAC (protease and lacunin) domain, present in ADAMTS2, 3, 6, 7, 10, 12, 14, 16, 17, 18, and 19, a Gon-1-like domain, found only in ADAMTS9 and 20, and a mucin domain, specific to ADAMTS7 and 12 (Figure 1).

To be active, the zymogen form of ADAMTS proteases has an N-terminal propeptide, which must be cleaved by proprotein convertases such as furin [3]. However, there are exceptions to ADAMTS9 and ADAMTS13, which stay active despite retention of the propeptide [4,5]. The propeptide cleavage may occur in the trans-Golgi network, or at the cell surface [6,7]. Most proteases of the ADAMTS family are modified by post-translational modifications, such as N- and O-glycosylation, the addition of chondroitin–sulfate chains, C-mannosylation of tryptophane residues, and O-fucosylation of serine/threonine residues in the thrombospondin type I repeat (TSR) [2]. These enzymes’ activities are mainly regulated by inhibitors, TIMP-3, α2-macroglobulin, and endocytosis mediated by the LRP-1 receptor leading to a decrease of functional and bioavailable ADAMTS in the cellular microenvironment [8,9,10,11].

Proteases of the ADAMTS family can be grouped according to their ancillary domains and then their substrate. They evolved likely by gene duplication that led to functional redundancy [12,13,14]. ADAMTS13 is the most well-known ADAMTS and is involved in von Willebrand factor proteolysis and hemostasis [15]. The proteoglycanases ADAMTS1, 4, 5, 8, and 15, as well as ADAMTS9 and 20, form a group that can cleave proteoglycans. More specifically, ADAMTS4 and ADAMTS5 were shown to be responsible for cartilage aggrecan destruction in arthritis. Among the procollagen N-propeptidases (ADAMTS2, 3, and 14), ADAMTS2 is well-characterized through its involvement in a rare human disorder, Ehlers-Danlos syndrome (EDS) type VIIC [16]. Recent data suggest that ADAMTS3 may not be an essential procollagen N-peptidase and it seems to have a role in lymphangiogenesis through activation of Vascular Endothelial Growth Factor-C (VEGF-C) [17]. ADAMTS6, 10, and 17 can be considered as proteases associated with fibrillin and fibronectin. This group’s function was discovered through the identification of mutations in Weill-Marchesani and Weill-Marchesani-like syndromes. Interestingly, certain peptides derived from cleaved ADAMTS may have a specific function [18].

In the ADAMTS superfamily, ADAMTS-Like (ADAMTSL) proteins are the products of distinct genes. They lack the catalytic domain as well as the propeptide and disintegrin-like domain present in all ADAMTS. They have a similar structure to the ancillary domains of ADAMTS. However, none of the ADAMTSLs display the exact composition of the ADAMTS ancillary domains: This suggests that ADAMTSLs interact with specific extracellular binding proteins. Most of the ADAMTSL proteins bind to microfibrils, such as ADAMTSL2, ADAMTSL4, ADAMTSL5, and ADAMTSL6 [19,20,21,22]. It has been shown that Drosophila Papilin, a basement membrane component closely structurally related to the ADAMTSL family, can act as a non-competitive inhibitor of bovine ADAMTS2 in vitro [23]. The data on *Drosophila* papilin and the structural similarities of ADAMTS proteases and ADAMTSLs suggest a potential functional link between these two types of proteins. The potential relationship between ADAMTS and ADAMTSL is an area that needs further research.

## 2. ADAMTS/ADAMTSL and TAA 

### 2.1. THSD4/ADAMTSL6 Mutations Responsible for TAA

The *THSD4* gene encodes the ADAMTSL6 protein. ADAMTSL6 is known to be a microfibril-associated extracellular protein without any catalytic activity. In E16.5 mouse embryos, ADAMTSL6 proteins were observed in the dermis, perichondrium, aortic wall, and all elastic tissues [21]. The ADAMTSL6 protein was also present in adult kidney artery walls and the mitral valve in the adult heart. All these data suggest that ADAMTSL6 is an ECM protein associated with elastic tissues in its fibrillary components. ADAMTSL6 interacts directly with fibrillin-1 (FBN1) at its N-terminal region and promotes early-stage FBN1 microfibril assembly [21]. This role was confirmed using transgenic mice where the *Adamtsl6* gene was overexpressed. 

The recent identification of *THSD4* as a new gene involved in TAA led us to consider a new function of this ADAMTSL protein [24]. The recent data support that *Thsd4*^+/−^ mice present progressive thoracic aortic dilation with disruption of elastic fibers and increased apoptosis of SMCs at eight months old. In human cellular models, the introduction of mutations in *THSD4*/*ADAMTSL6* leads to impairments of FBN1 microfibril assembly. In the aorta of the TAAD patients, the disorganization of the FBN1 microfibril network was confirmed and was associated with an increase of active TGF-β and overactivation of the TGF-β signaling pathway [24]. In the context of a tumor, ADAMTSL6 protein also indirectly regulates the TGF-β signaling pathway [25]. From these genetic and mouse model results, the role of ADAMTSL6 protein in the homeostasis of FBN1 in the aorta was highlighted. Another study suggests the possibility of using ADAMTSL6-mediated fibrillin-1 microfibril assembly as a therapeutic tool to rescue the cells of Marfan syndrome (MFS) [26]. Their findings highlight the improvement of the FBN1 network after administration of ADAMTSL6 and the following attenuation of the overactivation of TGF-β signaling.

### 2.2. Proteoglycanases Involved in TAA

Proteoglycans are one of the major groups of ADAMTS substrates. Their composition is a core protein associated with various glycosaminoglycans. In the vasculature, the proteoglycans are mainly expressed in endothelial cells and vascular smooth muscle cells of the intima and the media of the vessel wall. Due to their position in the ECM, they are involved in cell communication, signaling, and behavior. Despite the increase of proteoglycans as a hallmark of atherosclerosis plaques, recently, it was demonstrated that enhanced aggrecan and versican are also features of aneurysms [27]. The level of proteoglycans is regulated by proteoglycanases such as ADAMTS. Considering the importance of proteoglycans in the vessels, we will highlight the role of these enzymes in vascular diseases.

#### 2.2.1. ADAMTS1

ADAMTS1 protein was discovered in 1997 as the first member of the ADAMTS family. ADAMTS1 possesses a large catalog of substrates: aggrecan, versican, syndecan-4, TFPI-2, semaphorin 3C, nidogen-1, nidogen-2, desmocollin-3, dystroglycan, mac-2, gelatin (denatured collagen type I), amphiregulin, TGF-α, and heparin-binding EGF. This protease is expressed in many different types of cells and then in endothelial and vascular smooth muscle cells. 

ADAMTS1 is important in specific developmental processes. During heart development, ADAMTS1 participates in stopping the proliferation of ventricular cardiomyocytes by cleaving versican. These data indicate that ADAMTS1 may be the principal ADAMTS which mediates versican V1 cleavage during ventricular morphogenesis [28]. Fibulin-1 is the cofactor of this function in ADAMTS1 during cardiac ventricular development [29]. ADAMTS1 has also pro- and anti-angiogenic properties through either its nonenzymatic or catalytic functions, depending on the cellular context [30]. It was demonstrated that the C-terminus of ADAMTS1 and its associated three thrombospondin motifs promote anti-angiogenic properties by sequestering VEGF [31]. In prostate tumors, expression of ADAMTS1 was directly correlated to the diameter of blood vessels, thus proving a pro-angiogenic role of ADAMTS1. 

More recently, a study demonstrated that ADAMTS1 may play a role in the pathophysiology of aorta remodeling. Enhanced expression of *ADAMTS1* was correlated with the occurrence of aortic dissection in humans, and in mice under angiotensin II delivery [32]. These findings were confirmed via a study of *Adamts1* deficiency in mice. This mouse model showed a decreased susceptibility to β-aminopropionitrile-induced TAAD formation and rupture. Furthermore, *Adamts1*-deficient mice had no inflammatory cell infiltration from inhibiting inflammatory cytokine levels and macrophage migration. Altogether, ADAMTS1 might be a suitable candidate as a potential therapeutic target for TAA [33].

To be a good target, it is necessary to identify the molecules that regulate ADAMTS1 expression. Different molecules used in vascular remodeling (VEGF, angiotensin-II, interleukin-1β, and tumor necrosis factor α) enhance ADAMTS1 expression in two types of cells, endothelial and vascular smooth muscle cells. Intracellular signaling pathways are associated with enhanced ADAMTS1 expression by inducing pathological vascular remodeling. This upregulation is mediated by specific signal transduction pathways involving nuclear factor of activated T cells (NFAT) or CCAAT/enhancer-binding proteins (C/EBPβ), both transcription factors. These pathways are another possibility to target for treating vascular disease [34].

Oller et al. have shown that ADAMTS1 is an important mediator of vascular wall homeostasis and that its expression is decreased in individuals with MFS [35]. Opposite results were reported by other groups. An increase of ADAMTS1 protein and mRNA expression was shown in TAAD tissues compared to control aortic tissues [36,37]. In parallel, one of the ADAMTS1 substrates called versican was found to be more cleaved in TAAD tissues than in control aortic tissues. The authors suggested then that an increased level of ADAMTS1 may lead to TAA progression by degrading versican [36]. These conclusions were established using descending and not ascending aortas samples. These samples indicated a difference in ADAMTS1 regulation depending on the aorta type studied and/or the level of cleaved versican. 

Using an osmotic pump delivering angiotensin II for 28 days, untreated and angiotensin II-treated mice expressing heterozygously *Adamts1*, displayed aortic dilation [35]. A similar phenotype was observed when they knocked down directly *Adamts1* in SMCs of the adult mouse aortas using a lentivirus encoding an *Adamts1*-specific siRNA. 

Interestingly, the decrease of *Adamts1* levels in the mouse model conduced medial degeneration with elastic fibers breaks and excessive collagen and proteoglycan accumulation—two hallmarks of TAA. As observed in MFS mice, the increase of TGFβ activation was linked to media degeneration in aortic sections of Adamts1^+/−^, the third hallmark of TAA. It must be emphasized that no experiments have been made on *Adamts1^−/−^* due to perinatal lethality. However, this activation of TGFβ is secondary to aortic dilation in this *Adamts1*-deficient mouse model. The knockdown of ADAMTS1 is immediately followed by the induction of elastolysis driven by Matrix metalloproteinase-9 (MMP9) secreted from vascular smooth muscle cells (VSMCs) [35]. Elevated levels of aortic nitric oxide (NO) and nitric oxide synthase 2 (NOS2) were observed in *Adamts1*^+/−^ and in a mouse model of MFS. NOS2 inhibition was found to protect both types of mice from aortic dilation or medial degeneration [35]. This mouse model revealed the critical role of NO and ADAMTS1 in syndromic forms of TAAD such as Marfan syndrome. These results also established a link between FBN1, ADAMTS, and NO.

#### 2.2.2. ADAMTS4

ADAMTS4 is a well-known proteoglycanase and has angiomodulatory properties. Both pro-and anti-angiogenic activities have been attributed to ADAMTS4. The full length of ADAMTS4 promotes tumor angiogenesis and subsequently tumor growth. The C-terminal ancillary domain possesses both pro-and anti-angiogenic properties [38]. ADAMTS4 is expressed in endothelial cells [39].

ADAMTS4 was found to be highly expressed in SMCs and macrophages in the aortic wall of challenged mice who were administered a high-fat diet and angiotensin II infusion [40] ADAMTS4 expression was also enhanced in human aortic tissues [36]. Adamts4 deficient mice did not develop TAAD when administered a high-fat diet and angiotensin II infusion. Aortas in *Adamts4^−/−^* mice displayed reduced aortic diameter enlargement associated with a reduction of elastic fiber destruction. In addition, these mice had versican degradation, thus suggesting that *Adamts4* deficiency prevents aortic destruction and proteoglycan degradation [40]. ADAMTS4 seems to be involved in SMC apoptosis by directly interacting with and cleaving poly-ADP-ribose polymerase 1 (PARP-1), a nuclear protein that plays a role in DNA repair and cell survival. Surprisingly, ADAMTS4, as an enzyme, is involved in the turnover of ECM and has also a role inside the cell, in the nucleus, and induces SMC apoptosis. In the context of tumors, a role in apoptosis has been shown. The inactive ADAMTS4 enzyme or only its C-terminus domain inhibits melanoma growth and its angiogenesis associated with tumor cell apoptosis [38]. Its role in aortic aneurysms may be more connected to its ability to induce apoptosis rather than its versicanase function.

One study found that micro-RNA (miR)-126a-5p inhibited the upregulation of the *ADAMTS4* gene [41]. This miR-126a-5p was shown to be downregulated 18-fold in AAA samples of mouse aortas. In contrast, it was found that miR-126a-5p overexpression significantly improved survival rates and reduced aortic dilatation in Ang II-infused mice as well as reduced elastic fragmentation and ECM degradation. There are many indicators that ADAMTS4 is a new target for miR-126a-5p [41]; this miRNA seems to be a good therapeutic molecule candidate to modulate the expression of ADAMTS4 protein.

#### 2.2.3. ADAMTS5

ADAMTS5 is one of the main enzymes involved in proteoglycan cleavage. It was demonstrated that *ADAMTS5* mRNA and protein levels are reduced in TAAD tissues as well as in plasma [27,42]. Mice data using *Adamts5*-deficient mice point to the same trend, by developing TAA. Using Ang II as a model to induce TAAD, mice lacking the catalytic domain of ADAMTS5 protein by *Cre*-mediated exon excision (*Adamts5^Δcat^*) displayed a higher rate of enlargement of the ascending aorta. These results show a distinct and non-redundant role among ADAMTS4 and ADAMTS5. 

Versican plays a central role in the development of aortopathies, as it was found to be the most upregulated ECM protein in *Adamts5^Δcat^* mice. In parallel, a clear reduction of versikin, cleaved versican by specific ADAMTS, was observed in the aorta [43]. Low-density lipoprotein-related protein 1 (LRP1) is a regulator of ADAMTS5 by promoting its endocytosis. LRP1 is involved in aneurysm formation [44]. LRP1 protein levels were found to be lower in Ang II-treated aortas from *Adamts5^Δcat^* mice. *Adamts5* deficiency was discovered to also increase ADAMTS1 protein levels. *ADAMTS1* expression was not able to compensate for the absence of versican cleavage: this suggests that ADAMTS5 is a major versicanase [45]. To date, no data on ADAMTS4 expression levels are available in this mouse model. An additional mouse model was developed, the *Adamts5^−/−^* mouse model, and found aortic anomalies. The aortas of *Adamts5^−/−^* displayed aggrecan accumulation, thus confirming the key roles of aggrecan and versican in aortopathies. Results from these studies suggest that ADAMTS5 may have a critical role in normal aortic wall development [46].

#### 2.2.4. ADAMTS16

The exact function and the potential substrates of ADAMTS16 are unknown except for the substrate fibronectin [47]. The upregulation of ADAMTS16 was assessed using western blot on TAA aortic tissues [37]. It was suggested that enhanced expression of ADAMTS16 is involved in TAA progression. This finding was not surprising considering that ADAMTS16 has been associated with hypertension, one of the main causes of aortic aneurysm development [48]. Using a genetic rat model of hypertension with an *Adamts16* defect, the physiological observations established a linkage between *Adamts16* and blood pressure regulation. Heterozygous and homozygous *Adamts16* mutant rats presented a lower blood pressure along with a decrease in the aortic media layer thickness and pulse wave velocity. The pulse wave velocity is a measure of arterial stiffness. Furthermore, endothelial cell analysis showed the presence of elongated cilia in *Adamts16* deficient rats. Elongated cilia establish contact from the apical surface of the cell with the collagen in the extracellular matrix. Even though the function of ADAMTS16 is unknown, it may play an important role in vascular remodeling [48].

## 3. ADAMTS Linked to AAA

### 3.1. ADAMTS8

ADAMTS8 is an aggrecanase with low efficiency due to poor proteoglycans cleavage activity [49]. To date, this enzyme has been poorly investigated. Its expression profile shows expression in the heart and lungs. Its expression in the lung was correlated to its role in pulmonary arterial hypertension (PAH) [50]. In a mouse model using hypoxia to induce PAH, *ADAMTS8* was found to be enhanced in the lung, as well as in the pulmonary arterial SMC (PASMC) in PAH patients. In a vascular SMC-specific mouse model, *Adamts8^ΔSM22^*, under hypoxia-induced pulmonary hypertension it was found that PAH was reduced. Even if ADAMTS8 is part of the extracellular matrix, it was shown that ADAMTS8 has a role in intracellular signaling using PASMCs. The proliferation of PASMC was decreased when human recombinant ADAMTS8 was deleted [50]. The *ADAMTS8^−/−^* PASMC displayed increased phosphorylation of AMP-activated protein kinase/acetyl-CoA Carboxylase signaling. ADAMTS8 also may have endothelial function [50]. Secreted ADAMTS8 from PASMCs might be the mechanistic connection between PA endothelial cells and PASMCs in PAH pathogenesis. In this study, a therapeutic approach was tested using a high-throughput screening method. This screening revealed that mebendazole, a broad-spectrum antihelminthic which is indicated for the treatment of parasite infections, decreased ADAMTS8 expression and ameliorated PH in PH rat models [50]. 

In addition, a potential role of ADAMTS8 in the AAA was established [51]. Results from Farrell’s research found *ADAMTS8* to be downregulated in SMCs tissues collected from AAA patients, which elucidates its possible role in the pathology. All other AAA hallmarks were observed in patients’ tissues, such as lower elastin deposition and lysyl oxidase activity, or increased *FBN1* gene expression. 

Considering the absence of NO signaling in AAA, an optimal NO dosage treatment delivered via S-Nitrosoglutathione (GSNO) was used on SMCs from AAA patients [52]. The GSNO was found to ameliorate the stiffness of SMCs, decrease MMPs-2 and -9, and increase TIMP-1 release in AAA-SMC cultures. The study did not mention the impact of GSNO on *ADAMTS8* expression. An analysis of a genome-wide association study (GWAS) using data from the UK Biobank revealed novel genetic loci that may susceptible to the development of TAA and AAA [52]. In this GWAS research, *ADAMTS8* was an identified locus associated with an increased risk to develop AAA. 

### 3.2. ADAMTS9

ADAMTS9 is an actor in ciliogenesis [53]. This protease is also required for normal cardiovascular development. Heterozygous *Adamts9* mice were shown to have defects in the aortic wall, valvulosinus, and valve leaflets. These mice also displayed abnormal myocardial projections and a “spongy” myocardium, consistent with non-compaction of the left ventricle. These mutant mice heart anomalies were correlated with abnormal accumulation of versican and a decrease in cleaved versican compared to WT mice. These findings suggest a potentially important role for ADAMTS9 cleavage of versican in heart development [54]. In addition, ADAMTS9 expressed in vascular SMC may be a good candidate gene responsible for hereditary TAA. ADAMTS9 expression was identified as a marker of terminal AAA. Analysis of aortic wall tissues from patients with elective or emergency repair of ruptured AAA was used to establish the molecular changes leading up to AAA rupture [55]. *ADAMTS9* was found to only be upregulated in tissues from patients with an emergency repair of the abdominal aorta.

### 3.3. ADAMTS7 

It has been suggested that ADAMTS7 directly binds to the ECM protein named cartilage oligomeric matrix protein (COMP) in cartilage. COMP has been implicated in the pathogenesis of arthritis [56]. COMP was discovered to be expressed not only in skeletal tissue but also in the aorta. It has also been implicated in the attachment and haptotaxis of VSMCs [57]. In addition, COMP has also been associated with human atherosclerotic lesions, which suggests its potential importance during pathological ECM remodeling and VSMC migration [58,59]. ADAMTS7 has also been found to be involved in intimal thickening after vascular injury [60]. Altogether, these findings suggested that vascular remodeling is the consequence, at least in part, of ADAMTS7-dependent COMP degradation. 

Considering the potential role of ADAMTS7 in the pathogenesis of vascular atherosclerosis, ADAMTS7 may, therefore, be a potential therapeutic target in atherosclerosis. Using human tissues from aortic aneurysms (AA), it was shown that ADAMTS7 expression was significantly increased in the AA group compared to controls. At the same time, conversely, the COMP protein level was decreased in AA samples [61]. Even though the COMP cleavage by ADAMTS7 has not been able to be replicated using purified proteins, the ADAMTS7/COMP pathway is identified as a novel potential therapeutic target in human AA [61].

More recent studies have identified other potential substrates of ADAMTS7. One study showed that ADAMTS7 is implied in participating in the inhibition of endothelial cell proliferation and migration in vitro. Moreover, *Adamts7* null mice showed an excessive reendothelialization in injured arteries [60]. These findings suggest that ADAMTS7 protein may retard endothelium repair via thrombospondin-1 degradation. Interinstingly, some ADAMTS7 studies could not identify cleavage COMP products by terminal amine isotopic labeling of substrates (TAILS) [62,63].

Using this method, latent-TGFβ-binding protein 4 (LTBP4) was identified as an ADAMTS7 substrate [63]. Interestingly, LTBP4 is a component of microfibrils and can interact with FBN1 and fibulin-5, which are both involved in the formation of elastic fibers. *FBN1* mutations are associated with MFS which is characterized by the increased incidences of TAA. Lowered *fibulin-5* mRNA levels have been linked to patients with aortic dissection: decreased fibulin-5 may contribute to the pathogenesis of aortic dissection by impairing elastic fiber assembly [64]. Results from these studies support the potential link of ADAMTS7 as playing a role in LTBP4 cleavage in aortic aneurysms. Recently, an efficient inhibitor of ADAMTS7 was identified as being TIMP-4. TIMP-4 may be an interesting therapeutic molecule to further research its relational context to AA.

## 4. ADAMTSL2, ADAMTS10 and 17 Possibly Involved in Aorta Pathology

Some ADAMTSL family proteins are involved in rare disorders. These rare disorders, namely Acromelic dysplasias, include, among others, the Weill-Marchesani syndrome (WMS) and Geleophysic dysplasia (GD). Mutations in *ADAMTS10* (OMIM#277600) and *ADAMTS17* (OMIM#613195) have been identified in WMS cases, and mutations in *ADAMTSL2* (OMIM#231050) were associated with GD patients. These disorders share similar clinical features, such as short stature with shortened extremities, thick skin, and restricted joint mobility. These pathologies can also lead to cardiovascular defects that may be life-threatening conditions [65]. 

GD was first described in 1971 in two patients presenting, among others, short stature, short hands and feet, and limited joint mobility [66]. Homozygosity mapping in two unrelated non-consanguineous families and four unrelated consanguineous families found *ADAMTSL2* to be the causal gene of the recessive form of GD [19]. The first mutations were found either in the cysteine-rich module or in the TSR6 of the ADAMTSL2 protein [67].

Cardiovascular defects which affect GD patients manifest throughout their lifetimes. High mortality is observed due to cardiovascular/bronchorespiratory defects, making GD the most severe pathology among the Acromelic dysplasias [19]. In addition, pulmonary stenosis, mitral and aortic valves stenosis, and mild tricuspid regurgitation are also common cardiovascular manifestations found in GD [67,68,69,70]. Additionally, thickened ventricle walls associated with limited ejection fraction were found in GD cases, as well as PAH and hypertrophy of the papillary muscles [67,68,71].

More recently, in a case study, the defect in the *ADAMTSL2* gene was linked to dilatation of ascending aorta in a 48-year-old patient. This patient also presented aneurysms in the brachiocephalic artery and main pulmonary artery [71]. ADAMTSL2 is not known to be expressed in VSMC, but rather in other cardiovascular cells, such as cardiomyocytes and dermal blood vessels of human embryos [19]. *Adamtsl2* was found to be overexpressed in microvascular endothelial cells of ventricles in a murine model of transverse aortic constriction [72]. Using mass spectroscopy on healthy hearts and hearts presenting coronary diseases, ADAMTSL2 was found to be under-expressed in the unhealthy heart [73].

*Adamtsl2^−/−^* mice fail to survive postnatally and have cardiac developmental defects. This is likely due to lung anomalies associated with bronchial fibrillin microfibril accumulation [74,75]. In heart ventricles undergoing heart fibrosis due to heart failure, all ADAMTSLs mRNA were overexpressed except *ADAMTSL6*. *ADAMTSL2* was the most upregulated in cardiac fibroblasts [76]. This increase resulted in reduced TGFβ production and signaling pathway activation. Increased ADAMTSL2 levels inhibited myofibroblast differentiation through the inhibition of *ACTA2* expression. This, in turn, caused attenuation of important pro-fibrotic, phenotypic properties of cardiac fibroblasts, such as proliferation, migration, and contractility. Taken together, findings indicate that ADAMTSL2 may be a negative regulator of TGFβ in cardiac fibroblasts. Collective research implies that ADAMTSL2 regulates ECM deposition and TGFβ signaling and may, therefore, have an important role in cardiac fibrosis and heart failure.

Belonging to the group of genes involved in the Acromelic dysplasia spectrum, ADAMTS10 and 17 have not yet been associated with aneurysms, but they have been associated with cardiovascular defects. First described in 1932 by Weill [77] and later in 1939 by Marchesani [78], WMS has been suggested to be a “recessive condition” due to the parental consanguinity of affected patients. It was not until the early 21st century that homozygosity mapping in consanguineous WMS families highlighted the homozygous splicing and nonsense mutations in *ADAMTS10*. This gene encodes the ADAMTS10 protein and specifically impacts the metalloprotease domain of the protein [79,80]. Shortly after, the discovery of homozygous mutations in *ADAMTS17*, which encodes for the ADAMTS17 protein, has resulted in a new “Weill-Marchesani-like” phenotype [81,82]. Both genes lead to the recessive form of WMS.

Cardiovascular defects affecting WMS patients are mainly localized in the human heart. These defects include prolonged QTc (more than 0.46 s) with mitral valve prolapsus [83], hypertrophic obstructive cardiomyopathy, pulmonary stenosis with dysplastic valves [84,85], and small muscular ventricular septal defect [86]. To date, little is known about the exact role of ADAMTS10 and ADAMTS17 proteins in heart development and its homeostasis. ADAMTS10 is highly expressed in blood vessels and the human heart [6]. ADAMTS10 is specifically expressed in aortic valve leaflets, leading to what many think that malfunction ADAMTS10 proteins lead to mitral aortic prolapse [87]. ADAMTS17 is also expressed in the human heart [81]. ADAMTS10 and ADAMTS17 are also linked to fibrillin networks as ADAMTSL2 or ADAMTSL6. This suggests that these four proteins may have a role in the remodeling of the aorta where the fibrillin proteins are an important component of aortic elastic tissues.

## 5. ADAMTS18 Involved in Aorta Development

The role of ADAMTS18 in vascular development was researched by using an *Adamts18* knockout mouse model [88]. During the development of the mice, *Adamts18* mRNA was observed in cells surrounding the ascending aorta and carotid artery. *Adamts18* knockout mice presented malformations of the embryonic aortic arch and the carotid artery system, including disordered elastic fibers and reduced carotid blood pressure. They also presented hypoplasia of the thymus and the absence of the carotid body. These abnormalities could be explained by the deficiency of Adamts18, causing fibronectin accumulation and ECM remodeling. These phenomena would, thus, activate the Notch3 signaling pathway. This activation ends up affecting the differentiation of cranial neural crest cells into VSMC [88]. The repertoire of ADAMTS18 substrates needs to be further investigated to better understand the role of this enzyme in the aortic arch and carotid artery. FBN1 may be a substrate candidate. In the *Adamts18^−/−^*, an increased level of FBN1 was observed in a carotid artery with disorganized elastic fibers and lower blood pressure [88]. It has been shown that ADAMTS18 is an actor of FBN1 assembly in bronchial cells [89].

## 6. ADAMTS19 Implicated in Valve Development

Recent genetic data revealed a potential function of *ADAMTS19* in valve development. Loss of function *ADAMTS19* mutations have been identified as being responsible for non-syndromic heart valve disease [90]. Patients with heart valve disease represent only 2% of the general population. Using whole-exome sequencing, homozygous truncating nonsense variants in *ADAMTS19* were revealed in four affected cases of two distinct families. To understand the role of ADAMTS19 in valve development, a mouse model using the knockout first allele tagged with a lacZ reporter cassette was generated. The follow-up of the echocardiographic analysis displayed a progressive aortic valve disease at three months of age in 38% of the *Adamts19^KO^*^/*KO*^ mice. The abnormal valves presented disorganization of the ECM, which could be a consequence of the mutant metalloproteinase ADAMTS19. Mutants displayed higher proteoglycan content as well as thinner collagen fibers. Using lacZ staining, the expression of *Adamts19* was strongly detected in all four valves, and specifically in the valvular interstitial cells (VICs). Single-cell transcriptomic data suggested that there is a defect in the mechano-transduction pathway from VIC to endothelial cells, implying the Wnt signaling pathway.

## 7. Conclusions

The studies of human aortic tissues and the phenotypes of *Adamts*/*Adamtsl* gene knockout mouse models revealed the important roles of this family in aortic aneurysms. Multiple ADAMTS or ADAMTSL proteins seem to be involved in TAA as well as in AAA. However, from animal studies, it is possible to notice that there is no functional redundancy between these different ADAMTS (Table 1). It is certainly necessary to further develop our knowledge on their spectrum of substrates. The linkages between ADAMTS proteases and ADAMTSL proteins remain to be elucidated. For example, the first ADAMTSL, namely ADAMTSL6, which is associated with TAA, may have a cooperative role with ADAMTS1. The ultimate challenge will be to develop specific therapeutic approaches using inhibitors targeting the ADAMTS, for example, ADAMTS4—a pro aneurysm molecule.

## Figures and Tables

**Table 1:** *Adamts* associated with rodent phenotype traits.

Genotype	Treatment	Phenotype	Observations	References
*Tshd4^+/−^*	None	Thoracic aortic dilatation	Elastic fibers disruption, increased SMC apoptosis	[24]
*Adamts1^+/+^*	Ang II	TAD	High ADAMTS1 expression	[32]
*Adamts1^−/−^*	BAPN	Decreased susceptibility to TAAD	No inflammatory cell infiltration	[33]
*Adamts1^+/−^*	Ang II and none	TAA	Medial degeneration, elastic fibers breaks, collagen and proteoglycans accumulation, increased TGFβ activation, elevated aortic NO and Nos2	[35]
*Adamts4^+/+^*	Ang II and high-fat diet	AAD	High ADAMTS4 expression in aortic SMC and macrophages	[40]
*Adamts4^−/−^*	Ang II and high-fat diet	Decreased susceptibility to AAD	Reduced elastic fibers destruction and versican degradation	[40]
*Apoe^−/−^*	Ang II and miR-126a-5p	Reduction AAA	ADAMTS4 downregulation, improvement elastic fibers fragmentation and ECM degradation	[41]
*Adamts5* *^Δcat^*	AngII	TAAD	Ascending aorta enlargement, ECM versican upregulation, and versikin reduction, low LRP1 protein levels, high ADAMTS1 protein levels	[43]
*Adamts5* *^−/−^*	AngII	Aortic dilatation	Versican accumulation, aortic SMC loss, elastin degradation, abnormal adventitia	[46]
*Adamts16^+/−^* *Adamts16^−/−^*	None	Hypotension	Low blood pressure, media layer thickness decrease, pulse wave velocity decrease	[48]
*Adamts8* *^ΔSM22^*	Hypoxia	PAH reduction	PASMC proliferation decrease, AMPK upregulation, endothelial dysfunction, matrix metalloprotease activation	[50]
*Adamts9^+/−^*	None	Aortic wall defects, valve sinus and valve leaflets, abnormal myocardial projections, spongy myocardium	Abnormal versican accumulation, decrease in versican cleavage	[54]
*Adamts7^−/−^*	None	Excessive re-reendothelialization and reduced atherosclerosis	EC proliferation enhanced	[59,60,91]
*Adamtsl2^−/−^*	None	Post-natal death	Lung abnormalities associated with bronchial fibrillin microfibril accumulation, cardiac developmental defects	[74,75]
*Adamts18^−/−^*	None	Malformations in the aortic arch and carotid artery system, thymus hypoplasia, carotid body absence	Disordered elastic fibers, increased Fbn1 level, low blood pressure.	[88]
*Adamts19^−/−^*	None	Non-syndromic heart valve disease	ECM disorganization, high proteoglycan content, thin collagen fibers	[90]

AAD: Aortic aneurysm and dissection, AAA: Abdominal aortic aneurysm, BAPN: *β*-aminopropionitrile, ECM: extracellular matrix, TAA: Thoracic aortic aneurysm, TAAD: Thoracic aortic aneurysm and dissection, TAD: Thoracic aortic dissection, NO: Oxide nitric, NoS2: Oxide nitric synthase 2, PAH: Pulmonary arterial hypertension.

## Data Availability

Not applicable.
